# Sustainable Synthesis of Biogenic Hydroxyapatite from Eggshells: Effect of Processing Conditions and Comparative Analysis by FTIR, XRD, and SEM

**DOI:** 10.1021/acsomega.6c00153

**Published:** 2026-07-07

**Authors:** Rômulo D. Correa, Maick S. Pimentel, Marcelino J. dos Anjos, Eustáquio de S. Baeta Junior, Letícia dos S. Aguilera

**Affiliations:** † Polytechnic Institute, Graduate Program in Materials Science and Technology—Nova Friburgo, Rio de Janeiro State University, Rio de Janeiro 28625-570, Brazil; ‡ Institute of Physics, Graduate Program in Physics— Rio de Janeiro, Rio de Janeiro State University, Rio de Janeiro 20550-013, Brazil; § Rio de Janeiro State University, Faculty of Engineering, Graduate Program in Mechanical Engineering—Rio de Janeiro, Rio de Janeiro State University, Rio de Janeiro 20550-013, Brazil

## Abstract

Although eggshell-derived calcium sources have been widely explored for hydroxyapatite (HAp) synthesis, the minimum processing conditions required to obtain crystalline and high phase-purity HAp under comparable wet-chemical routes remain insufficiently defined. In this study, six wet-chemical synthesis methodologies were systematically compared using CaO derived from chicken eggshells as the calcium source, while maintaining equivalent chemical conditions and varying only key processing parameters, particularly the presence or absence of moderate heating. The synthesized materials were characterized by Fourier transform infrared spectroscopy (FTIR), X-ray diffraction with Rietveld refinement (XRD), and scanning electron microscopy. Quantitative XRD analysis revealed that nonheated routes resulted in incomplete conversion, with the persistence of intermediate calcium phosphate phases, whereas methodologies employing moderate heating promoted enhanced crystallinity and phase purity. Among the investigated routes, Methodology 6, conducted at 70 °C, exhibited the most advanced structural development, yielding predominantly hydroxyapatite, with approximately 88% hexagonal HAp and 11% monoclinic HAp, nanometric crystallite sizes (≈12–33 nm), and minimal residual Ca­(OH)_2_.

## Graphical abstract

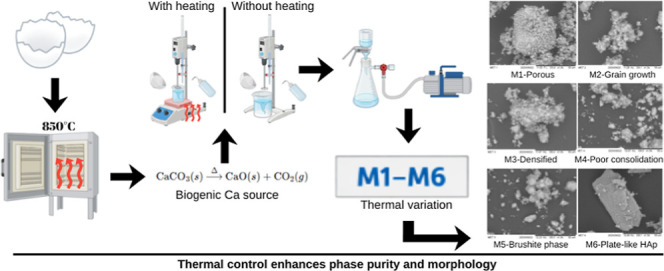

## Introduction

Hydroxyapatite (HAp, Ca_10_(PO_4_)_6_(OH)_2_) is a bioactive calcium phosphate widely employed in bone regeneration due to its composition and bioactivity similar to those of mineralized human tissue.^1^ At the same time, “green” routes that exploit calcium-rich waste, such as eggshells, have gained prominence for reducing costs and environmental impacts, providing an abundant CaCO_3_ precursor for HAp synthesis.^2,3^

Several wet-chemical approaches have been reported to convert eggshell-derived calcium precursors into HAp, particularly conventional aqueous precipitation and hydrothermal processing, which enable control over stoichiometry, crystallite size, crystallinity, and morphology.^4^ Previous studies using precipitation-based routes have demonstrated the feasibility of obtaining HAp from eggshell-derived calcium sources, whereas hydrothermal methods have been shown to enhance crystallinity and morphological control under controlled temperature and pressure conditions.^5^ ^,^

In addition to direct HAp synthesis, several studies use eggshell calcination to convert CaCO_3_ into CaO, which acts as a reactive calcium precursor in the wet-chemical synthesis. The calcination temperature directly influences the carbonate decomposition efficiency, crystallinity, and phase stability of the resulting precursor. Furthermore, the exposure of CaO to atmospheric moisture may promote its hydration to Ca­(OH)_2_, affecting the structural and spectroscopic characteristics of the material. Recent studies have highlighted the importance of controlling calcination conditions to obtain reproducible HAp with high structural quality.^6,7^ Recent studies have also shown the reuse of eggshell waste in other bioceramics (e.g., bioactive calcium silicates) and in functional sorbents, reinforcing the relevance of this residue as a technological raw material.^8,9^

Multitechnique characterization is indispensable for correlating synthesis route and performance. FTIR spectroscopy enables the identification of functional groups such as PO_4_^3–^, OH^–^, and CO_3_^2–^, as well as monitoring impurities/carbonation; XRD provides insights into phase composition and crystallinity; and Rietveld refinement allows quantitative phase analysis and lattice parameter determination with high metrological accuracy.^10,11^ Scanning electron microscopy (SEM), in turn, elucidates particle morphology and size distribution—factors that directly affect bioactivity. Recent reviews and studies on eggshell-derived HAp corroborate this integrated characterization approach.^2,3^

Despite the progress in HAp synthesis routes, challenges remain regarding the production of materials with high phase purity and morphological control from the natural precursors. Residual impurities, variations in raw material composition, and susceptibility to carbonation during processing may compromise the final properties of the biomaterial.^2,6^

Although several studies have successfully reported the synthesis of eggshell-derived hydroxyapatite with high crystallinity and phase purity, a direct comparison among different wet-chemical routes remains limited. In most reported works, synthesis parameters such as calcination temperature, calcium precursor composition, phosphate source, pH, aging time, reaction temperature, and postsynthesis heat treatment are varied simultaneously. As a result, it is difficult to determine whether the final phase purity and crystallinity are primarily governed by the synthesis route itself or by secondary processing variables. Therefore, systematic studies performed under equivalent chemical conditions are still required to clarify how different wet-chemical methodologies affect the formation of crystalline and phase-pure HAp from egg-shell-derived calcium precursors.

Although several studies have reported the successful synthesis of eggshell-derived HAp with high crystallinity and phase purity, these results are often associated with specific synthesis conditions, such as calcination temperature, pH control, aging time, and postsynthesis thermal treatment. Consequently, direct comparison among different wet-chemical methodologies remains limited since multiple experimental parameters are simultaneously modified across the studies reported in the literature. In this context, comparative analyses conducted under equivalent chemical conditions, using the same calcium precursor source and comparable synthesis parameters, become relevant to isolating the influence of the synthesis methodology on HAp formation.^7,8^

Therefore, the present study systematically compares six wet-chemical synthesis methodologies while maintaining constant chemical composition (fixed Ca/P molar ratio and identical reactant concentrations) and varying exclusively key processing parameters, particularly the presence or absence of moderate heating during precipitation. By isolating thermal input as the central variable, this approach allows a direct and reproducible evaluation of its role in phase formation, crystallinity development, and microstructural evolution in eggshell-derived hydroxyapatite. In doing so, this work organizes fragmented findings from the literature and establishes clearer processing–structure relationships for sustainable HAp production from biogenic CaO.

From a social and environmental perspective, this work adds value to agro-industrial waste and aligns the research with the United Nations 2030 Agenda for Sustainable Development. The use of eggshells as a calcium source contributes to waste reduction and circular economy practices (SDG 12, target 12.5), promotes cleaner and safer synthesis routes (SDG 3, target 3.9), and supports low-impact, innovation-driven approaches in biomaterials processing (SDG 9, target 9.4). Overall, the study integrates circularity, sustainable innovation, and environmental mitigation, reinforcing the social relevance of producing biomaterials from renewable resources.

Despite the large number of studies devoted to the synthesis of hydroxyapatite from eggshells, a significant limitation still persists in the literature because of the lack of systematic comparisons conducted under equivalent chemical conditions. In many reports, different routes are evaluated in isolation or with multiple parameters varying simultaneously, which hinders the objective identification of the minimum conditions required to suppress intermediate phases and obtain hydroxyapatite with a high phase purity and crystallinity.

The products obtained from the synthesis routes were investigated using FTIR, XRD with Rietveld refinement, and SEM, aiming to map how synthesis conditions influence phase composition, crystallinity, microstructure, and consequently the potential application as a biomaterial.

## Materials and Methods

White chicken eggshells (Gallus gallus domesticus) were collected from domestic waste, washed under running water, manually cleaned to remove the inner membranes, and dried at room temperature for 72 h. The material was subsequently calcined in a muffle furnace under an air atmosphere at 850 °C, using a heating rate of 10 °C/min and an isothermal dwell time of 2 h, promoting the complete decomposition of calcium carbonate (CaCO_3_) and the formation of calcium oxide (CaO). After calcination, the material was naturally cooled to room temperature and stored in sealed containers until further use.

The syntheses were carried out at the Chemistry Laboratory of the Polytechnic Institute of Rio de Janeiro (IPRJ) with the aim of evaluating the impact of thermal synthesis conditions on the structural properties of the obtained material. Phosphoric acid (H_3_PO_4_), ammonium hydroxide (NH_4_OH), distilled water, and standard laboratory glassware were used for the synthesis of hydroxyapatite (HAp). The synthesized powder was washed with distilled water during vacuum filtration (MASTERCOOL, model 90067.220) and dried at 110 °C in a sterilization oven (BRASDONTO) for 12 h.

The complete processes of eggshell preparation, CaO production, and different HAp synthesis routes are presented in detail in this article, highlighting the methodological differences and processing variables. The calcination of eggshells was performed in a muffle furnace (FORTELAB ME1700/20) at the High-Temperature Furnace Laboratory (LFAT) of the Polytechnic Institute of the State University of Rio de Janeiro (IPRJ/UERJ).

### General Protocol for the Synthesis of Hydroxyapatite (HAp) from CaO Obtained from Eggshells

The synthesis of hydroxyapatite (HAp) was carried out from CaO obtained by eggshell calcination. Initially, the amount of CaO indicated for each methodology was dispersed in distilled water under stirring (mechanical or magnetic, depending on the methodology) for 30 min. Subsequently, an H_3_PO_4_ solution P.A. (Êxodo Científica, 85%) (either pure or diluted in distilled water, depending on the methodology) was added dropwise using a buret, at a rate of one drop every 4 s. During synthesis, phosphoric acid was added under constant stirring without prior control of the reaction medium pH. After complete acid addition, the pH of the suspension was measured using pH indicator paper (Macherey-Nagel) and adjusted to a final value of 11 by controlled addition of analytical-grade ammonium hydroxide (NH_4_OH) solution P.A. (VETEC, 28–30%). The same pH verification and adjustment procedure was applied to all methodologies, ensuring experimental consistency among the synthesis routes. The system was kept under stirring for an additional period (30 or 60 min, depending on the methodology) for solution aging. At the end of the process, the obtained material was filtered, washed with distilled water, and dried at 110 °C in a sterilization oven (BRASDONTO) for 12 h.

In all synthesis methodologies, the calcium-to-phosphorus (Ca/P) molar ratio was fixed at 1.67, corresponding to the stoichiometric value of hydroxyapatite (Ca_10_(PO_4_)_6_(OH)_2_). The Ca/P ratio was calculated on the basis of the effective mass of CaO obtained from eggshell calcination and the molar concentration of the phosphoric acid solution. This ratio was kept constant in all synthesis routes to ensure experimental reproducibility and to enable a direct comparison among the evaluated methodologies.

Due to the high reactivity and hygroscopic nature of eggshell-derived CaO, partial hydration and atmospheric carbonation during handling and storage are unavoidable. In this study, such effects were considered inherent to the use of biogenic CaO and were treated consistently across all methodologies through standardized storage and handling procedures. Consequently, any contributions from these processes did not compromise the comparative analysis among the evaluated synthesis routes.

### Characterization Techniques

#### FTIR

The HAp syntheses were analyzed at the Biomaterials Laboratory of the Polytechnic Institute of the State University of Rio de Janeiro (IPRJ/UERJ) by infrared spectroscopy using an attenuated total reflectance (ATR) accessory (Pike Miracle Single Reflection ATR, Pike Technologies) coupled to a PerkinElmer Frontier FTIR spectrometer with a sampling accessory. The analysis was carried out under standard conditions, with a resolution of 4 cm^–1^ and 256 scans, over the spectral range of 4000–700 cm^–1^. All spectra were plotted using SciDAVis software.

#### XRD

X-ray diffraction was performed using a commercial benchtop D2 Phaser diffractometer (BRUKER) located at the Laboratory of Electronic Instrumentation and Analytical Techniques (LIETA), UERJ. The equipment is fitted with a fast one-dimensional (1D) LYNXEYE detector. A copper anode X-ray source (Cu–Kα1 = 1.541 Å/8.047 keV) with a nickel filter was used, operating at a maximum power of 300 W (30 kV × 10 mA), with an angular step size of 0.002° and acquisition time of 5 s, within the range of 5° to 90° in 2θ under rotation (15 rpm).

Refinement of the results was carried out using the Rietveld method^12,13^ implemented in TOPAS-Academic software, version 7.2, which applies fundamental parameters for structural refinement by comparing the experimental diffractogram to a mathematical model that accounts for structural, instrumental, and diffraction conditions. Indexing cards were obtained from the Materials Project database (https://next-gen.materialsproject.org/). The GOF (goodness of fit) parameter was used to assess refinement quality, with values below 4.5 considered acceptable for linear detectors. The blue line represents the experimental data, the red line corresponds to the calculated profile, and the gray line shows the difference between the two.

#### SEM

Scanning electron microscopy (SEM) micrographs were obtained at the Polymer Technology Laboratory (TECPOL) using a Hitachi TM3000 (Hitachi High-Technologies, Japan), a benchtop microscope operated under low-vacuum conditions. The equipment was operated at accelerating voltages of 5 kV and 15 kV, adjusted according to the required resolution and contrast of the sample. The system allows magnifications from 15× to 30,000× and accommodates specimens up to 70 mm in diameter and 50 mm in thickness, requiring no complex preparation. For this study, powders and sintered bodies were directly mounted on aluminum stubs using conductive carbon tape. Images were acquired at different magnifications (100× to 5000×) to evaluate both overall morphology and microstructural features of the particles.

## Results and Discussion

### Calcined Eggshell Analysis

The X-ray diffraction (XRD) analysis of the calcined eggshells revealed the predominance of calcium hydroxide (Ca­(OH)_2_, portlandite), with an estimated mass fraction of 94.05%, along with approximately 5.95% adsorbed or structurally incorporated water (Figure 1). The Rietveld refinement yielded a GOF of 1.74, indicating a reliable adjustment for the data obtained using a linear detector.

**Figure 1:**
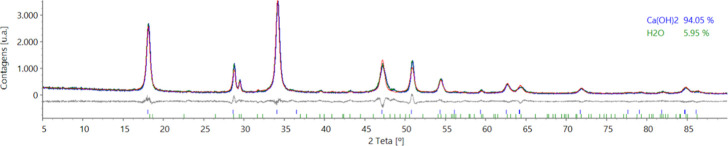
XRD diffractogram of the sample obtained from eggshell calcination, showing the predominant formation of calcium hydroxide (Ca­(OH)_2_).

The preferential formation of Ca­(OH)_2_, instead of calcium oxide (CaO), is attributed to the spontaneous hydration of CaO during or after calcination. This phenomenon has been extensively reported in the literature under air-exposure conditions due to the high hygroscopicity of CaO.^14–16^

Despite the absence of pure CaO, Ca­(OH)_2_ represents an effective precursor for the synthesis of hydroxyapatite (HAp). Recent studies employing eggshell-derived precursors in wet-chemical and microwave-assisted methods have reported the production of highly pure and bioactive powders.^2,5,17^

Cu Kα radiation (λ = 1.5406 Å); 2θ scanning range of 10–80°; step size of 0.02°; counting time of 1 s per step; measurements performed at room temperature (25 °C).

The FTIR spectrum of calcined eggshells (Figure 2) indicates that the sample is predominantly composed of Ca­(OH)_2_, but with variations in band intensity and definition. The strong O–H stretching at 3640 cm^–1^ (region D) confirms partial hydration of the oxide, consistent with the high reactivity and moisture exposure of biogenic CaO.^17,18^ Carbonate bands at 1450–1400 cm^–1^ and ∼875 cm^–1^ (regions A and C) reveal partial carbonation, either from atmospheric CO_2_ or residual carbonate not decomposed during calcination, with higher intensities reflecting greater susceptibility to carbonation.^2,19^

**Figure 2:**
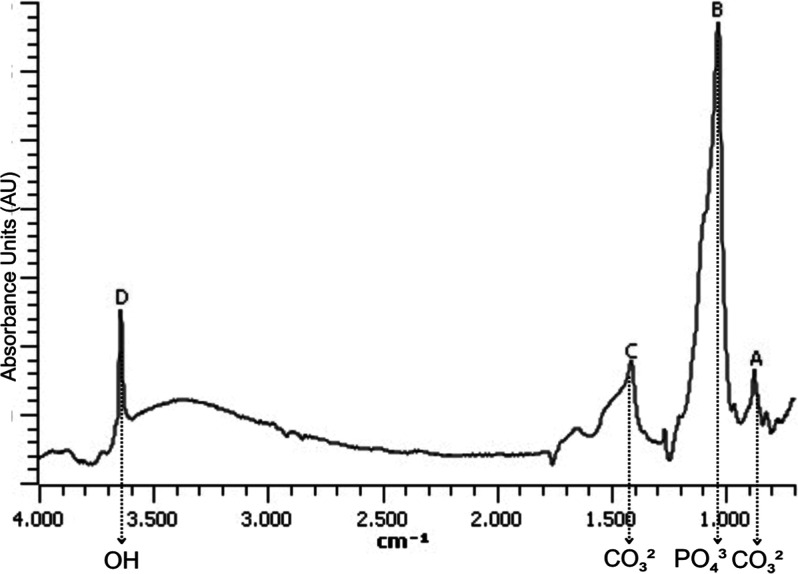
FTIR spectrum of CaO derived from calcined eggshells, highlighting the intense OH^–^ band at 3640 cm^–1^, the carbonate bands at ∼1450–1400 and 875 cm^–1^, and peaks associated with residual phosphate impurities.

A peak near 1010 cm^–1^ (region B) is attributed to residual mineral impurities such as traces of calcium phosphate or other substituted salts, commonly found in eggshell-derived CaO and known to facilitate subsequent HAp nucleation.^17,20^ Overall, the spectrum shows that the material retains functional impurities and a degree of hydration that can act as active sites for hydroxyapatite formation, as reported in thermally assisted and microwave-assisted routes.^2,19^

Spectral range: 4000–400 cm^–1^; spectral resolution: 4 cm^–1^; number of scans: 32; ATR mode (diamond crystal); measurements performed at room temperature (25 °C).

The SEM micrographs of CaO obtained from eggshell calcination revealed a typically irregular morphology, consisting of agglomerates of particles with rough contours and a heterogeneous size distribution.

At intermediate magnification of 1.5 k× (Figure 3A), regions with porous and irregular textures can be identified, suggesting the presence of residual micropores originating from the thermal decomposition of CaCO_3_. This microstructure is consistent with the hygroscopic behavior of CaO, which tends to react with ambient moisture, favoring the nucleation of secondary particles.^5^

**Figure 3:**
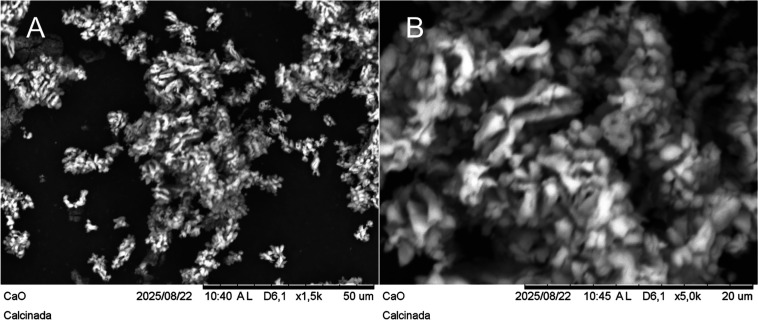
SEM micrographs of CaO obtained from eggshell calcination at different magnifications: (a) 1500×, revealing porous texture and particle coalescence; and (b) 5000×, highlighting submicrometric particles adhered to larger blocks.

At higher magnification of 5 k× (Figure 3B), smaller particles adhered to the surface of larger blocks are evident, indicating a hierarchical microstructure composed of both primary crystals and secondary agglomerates. This aspect is directly related to the high reactivity of CaO, since the large exposed surface area favors its interaction with atmospheric water and CO_2_.^17^

Overall, the SEM results confirm that CaO derived from eggshells exhibits an irregular and highly reactive morphology, consistent with observations in recent studies employing biogenic waste as a calcium source for hydroxyapatite synthesis.^21^ This characteristic is fundamental for the subsequent HAp synthesis process as it ensures high ionic availability and good precursor reactivity.

Accelerating voltages: 5–15 kV; magnifications: 1500×, and 5000×; secondary electron imaging mode; measurements performed at room temperature (25 °C).

### XRD Analysis and Rietveld Refinement of Methodologies 1 to 6

Figure 4 shows the XRD diffractograms and Rietveld refinements for samples obtained from all methodologies (M-1 to M-6), enabling a direct comparison of crystalline phase evolution as a function of thermal treatment. Refinements were performed in TOPAS-Academic v7.2 using the fundamental parameters approach and crystallographic cards from The Materials Project database.^22–28^

**Figure 4:**
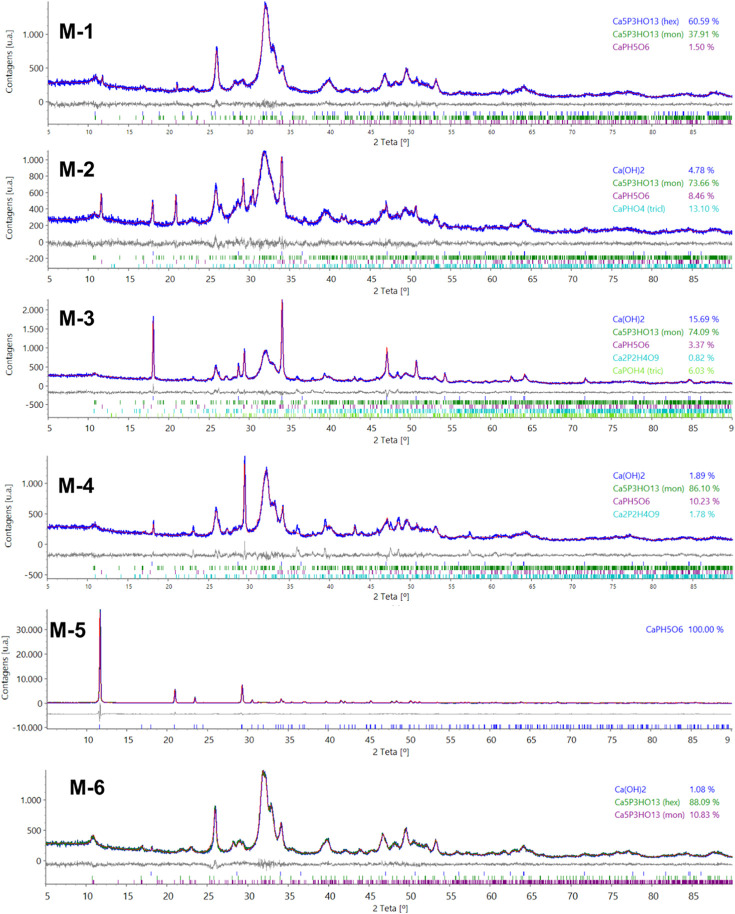
XRD patterns and Rietveld refinements of hydroxyapatite samples synthesized by Methodologies 1–6. Thermal treatment promotes higher phase purity and crystallinity, favoring the formation of hydroxyapatite and suppressing intermediate calcium phosphate phases. Cu Kα radiation (λ = 1.5406 Å); 2θ scanning range of 10–80°; step size of 0.02°; counting time of 1 s per step; measurements performed at room temperature (25 °C).

The diffraction pattern of Methodology 1 (M-1) reveals monoclinic hydroxyapatite (HAp) as the major phase (∼73.66%), accompanied by Ca­(OH)_2_ (∼4.78%), anhydrous dicalcium phosphate (CaHPO_4_, ∼8.46%), and brushite (CaHPO_4_·2H_2_O, ∼13.01%). The GOF (1.13) indicates good refinement quality. The coexistence of HAp and precursor calcium phosphates suggests that the reaction did not achieve full conversion, mainly due to the absence of thermal energy.^17,29^ Brushite and CaHPO_4_ are well-known intermediates in wet-chemical HAp synthesis, remaining stable when maturation time or temperature are insufficient.^8^ Residual Ca­(OH)_2_ likely arises from incomplete reaction with phosphate or hydration of CaO during handling.^5^

Methodology 2 (M-2) presents predominantly hexagonal HAp (∼60.59%) and monoclinic HAp (∼37.91%), with only ∼1.50% brushite. The GOF (1.10) demonstrates excellent refinement consistency with the crystallographic model. Compared with M-1, Methodology 2 shows higher conversion to crystalline HAp and lower intermediate-phase content, confirming that moderate heating during precipitation promotes HAp formation.^30^ The relative proportions of hexagonal and monoclinic forms can influence solubility, stability, and mechanical performance.^29^ In this context, the hexagonal HAp phase is generally considered the thermodynamically most stable polymorph, whereas the monoclinic form is associated with metastable structural arrangements arising from local disorder in the hydroxyl columns. The coexistence of both polymorphs suggests a partially ordered structure that may exhibit slightly increased solubility compared to fully hexagonal HAp, while still maintaining adequate structural stability.^10,19^ The marked reduction in brushite in M-2 aligns with studies reporting accelerated conversion at 60–90 °C.^5,17^

Methodology 3 (M-3), performed at 90 °C, shows predominant hexagonal HAp with minor fractions of Ca­(OH)_2_, CaHPO_4_, brushite, and a small amount of Ca_2_P_2_O_7_·2H_2_O. The GOF (≈1.34) is acceptable for multiphase systems analyzed using linear detectors.^31^ Crystallographic cards used include MP-41472 (hexagonal HAp), MP-721624 (monoclinic HAp), MP-23879 (Ca­(OH)_2_), MP-24389 (brushite), MP-1194747 (CaHPO_4_), and MP-746426 (Ca_2_P_2_O_7_·2H_2_O).^32^ Although the Ca/P molar ratio was maintained at the stoichiometric value of 1.67 in all methodologies, the results demonstrated that HAp formation was also strongly influenced by the characteristics of the calcium precursor and the reaction conditions employed. Therefore, the observed phase purity depends not only on the nominal reagent proportion but also on factors such as precursor hydration state, temperature, aging time, reaction kinetics, and carbonate incorporation during synthesis.^7,8^ The persistence of Ca­(OH)_2_ and intermediate calcium phosphate phases in some methodologies reinforces the importance of these parameters in controlling phase evolution and crystallinity during eggshell-derived HAp synthesis.^17,33,34^

Methodology 4 (M-4), synthesized without heating, yielded monoclinic HAp (86.10%), CaHPO_4_ (∼10.23%), Ca­(OH)_2_ (∼1.89%), and Ca_2_P_2_O_7_·2H_2_O (∼1.78%). The GOF (1.39) indicates satisfactory refinement quality but lower than that of the heated routes. The presence of pyrophosphate phases suggests partial phosphate condensation under cold conditions.^8,31^ The higher CaHPO_4_ content and the absence of the hexagonal HAp form reinforce the importance of thermal energy for full transformation into well-crystallized HAp.^5,21^ Comparatively, M-3 clearly outperforms M-4, confirming the necessity of heating to suppress intermediate phases.

From a structural standpoint, the absence of the hexagonal HAp phase in nonheated methodologies (M-1, M-4, and M-5) reflects insufficient atomic mobility for complete lattice reorganization. Such structural disorder is commonly associated with lower crystallographic stability and increased solubility, which may be undesirable for applications requiring long-term structural integrity.^18,35^

The contrast becomes most evident in methodologies 5 and 6. Methodology 5 (M-5), performed without heating, produced exclusively brushite (100%) with no HAp reflections. The GOF (2.38) remains acceptable for highly oriented brushite patterns but reflects a strong preferred orientation. This confirms that the synthesis was interrupted at an intermediate stage, lacking the necessary thermal input for HAp formation.^35^ Brushite persistence is widely reported in low-temperature wet-chemical routes.^17^

In sharp contrast, Methodology 6 (M-6) exhibited excellent refinement quality (GOF = 1.14) and demonstrated almost complete conversion to hydroxyapatite: hexagonal HAp (88.09%), monoclinic HAp (10.83%), and a small residual Ca­(OH)_2_ fraction (∼1.08%). Crystallite sizes were estimated as 12.29 nm (hexagonal) and 33.29 nm (monoclinic), values consistent with wet-chemical HAp subsequently matured under heating.^12,36^ The predominance of hexagonal HAp in M-6 indicates a more stable apatite structure, while the smaller monoclinic fraction may contribute to controlled surface reactivity. From a biomedical perspective, this balance between structural stability and moderate solubility is considered advantageous, as it may promote bioactivity and ion exchange at the interface without compromising the long-term integrity of the material.^8,17^ The refinement used crystallographic data MP-23879 (Ca­(OH)_2_), MP-41472 (hexagonal HAp), and MP-721624 (monoclinic HAp).

In summary, the XRD and Rietveld results from all methodologies (M-1 to M-6) demonstrate a coherent trend: thermal treatment is essential for suppressing intermediate phases (brushite, CaHPO_4_, Ca_2_P_2_O_7_·2H_2_O) and enabling full conversion into high-crystallinity HAp. Cold routes (M-1, M-4, M-5) result in incomplete transformation and higher precursor-phase content, whereas heated routes (M-2, M-3, M-6) progressively enhance conversion, crystallinity, and structural purity. Methodology 6 achieves the most advanced structural development, producing HAp suitable for biomedical applications (see Table 1).^31^

**Table 1:** Specific Differences between Synthesis Methodologies

methodology	stirring type	heating	aging time	total volume (mL)	CaO mass (g)
1	Mechanical	None	30 min	50	4.68
2	Mechanical	90 °C	30 min	50	4.68
3	Magnetic	90 °C	1 h	250	10.00
4	Magnetic	None	1 h	250	10.00
5	Mechanical	None	1 h	50	4.68
6	Mechanical	70 °C	1 h	50	4.68

Table 2 presents the refinement data for identified phases. This result clearly demonstrates that heating is decisive for obtaining crystalline, high-purity HAp with well-defined structures and crystallite sizes in the nanometer range.

**Table 2:** Crystalline Phases Identified by XRD and Rietveld Refinement

methodology	identified phases (wt %)	crystallite size (nm)	GOF	MP cards used
Ca(OH)_2_	Ca(OH)_2_, H_2_O (traces)—16.35%	–	1.74	MP-23879, MP-696735
M1	monoclinic HAp (73.66%), CaHPO_4_·2H_2_O (13.01%), CaHPO_4_ (8.46%), Ca(OH)_2_ (4.78%)	–	1.13	MP-23879, MP-1194747, MP-24389
M2	hexagonal HAp (60.59%), monoclinic HAp (37.91%), CaHPO_4_·2H_2_O (1.50%)	–	1.10	MP-23879, MP-41472, MP-721624, MP-24389
M3	Predominant hexagonal HAp (∼74.1%) with Ca(OH)_2_ (∼15.7%), brushite (CaHPO_4_·2H_2_O), CaHPO_4_ (anhydrous), Ca_2_P_2_O_7_·2H_2_O (traces)	–	1.34	MP-41472, MP-721624, MP-23879, MP-24389, MP-1194747, MP-746426
M4	monoclinic HAp (86.10%), CaHPO_4_ (10.23%), Ca(OH)_2_ (1.89%), Ca_2_P_2_O_7_·2H_2_O (1.78%)	–	1.39	MP-23879, MP-721624, MP-24389, MP-746426
M5	CaHPO_4_·2H_2_O (Brushite) (100%)	65.68	2.38	MP-24389
M6	hexagonal HAp (88%), monoclinic HAp (11%), Ca(OH)_2_ (1%)	12.29 (hex.), 33.29 (mon.)	1.14	MP-23879, MP-41472, MP-721624

### Comparative FTIR Analyses of Methodologies 1–6

The bands listed (A–I) are consistent with stoichiometric or slightly carbonated hydroxyapatite commonly produced by wet-chemical routes from Ca-rich biogenic residues.^21,29,30,34^ The ν_1_/ν_3_(PO_4_^3–^) modes (∼962–1052 cm^–1^; B–D), the carbonate ν_2_/ν_3_(CO_3_^2–^) vibrations (∼872–875 and 1416–1490 cm^–1^; A, E, F), the H–O–H bending (∼1640–1650 cm^–1^; G), and the OH^–^ stretching region (∼3.5–3.6 cm^–1^; H, I) confirm the presence of carbonated HAp.^37^ The shoulder at 1091–1119 cm^–1^ (D), associated with ν_3_ splitting, indicates the degree of local lattice order.^17^

The presence of carbonate bands at ∼872–875 cm^–1^ and 1416–1490 cm^–1^ indicates partial CO_3_^2–^ substitution within the apatite lattice, commonly classified as B-type substitution when carbonate replaces phosphate groups. Such carbonate incorporation is characteristic of wet-chemical synthesis routes conducted under atmospheric conditions and reflects local chemical disorder rather than long-range crystallinity changes.^21,29,30^

When comparing the six methodologies (Figure 5), all spectra display the characteristic profile of carbonated HAp; however, peak definition varies according to the presence or absence of heating. Methodology 2 shows sharper phosphate bands—especially band C—and a more distinct OH^–^ stretching (∼3570 cm^–1^), indicating improved crystallinity and more efficient incorporation of structural hydroxyls compared to Methodology 1. In M-1, the broader OH^–^ band suggests a higher occurrence of anionic vacancies or partial CO_3_^2–^ substitution.^34^ Despite similar carbonate intensities (bands E and F), both methods exhibit atmospheric carbonation typical of open-air precipitation.^5^ Variations in the intensity and definition of the OH^–^ stretching band (∼3570 cm^–1^) suggest differences in hydroxyl occupancy within the apatite structure. Broader or less intense OH^–^ bands, as observed in nonheated methodologies, indicate hydroxyl deficiency and anionic vacancies, often compensated by carbonate incorporation. These features reflect local structural imperfections that are not directly resolved by XRD analysis.^31,34,38^

**Figure 5:**
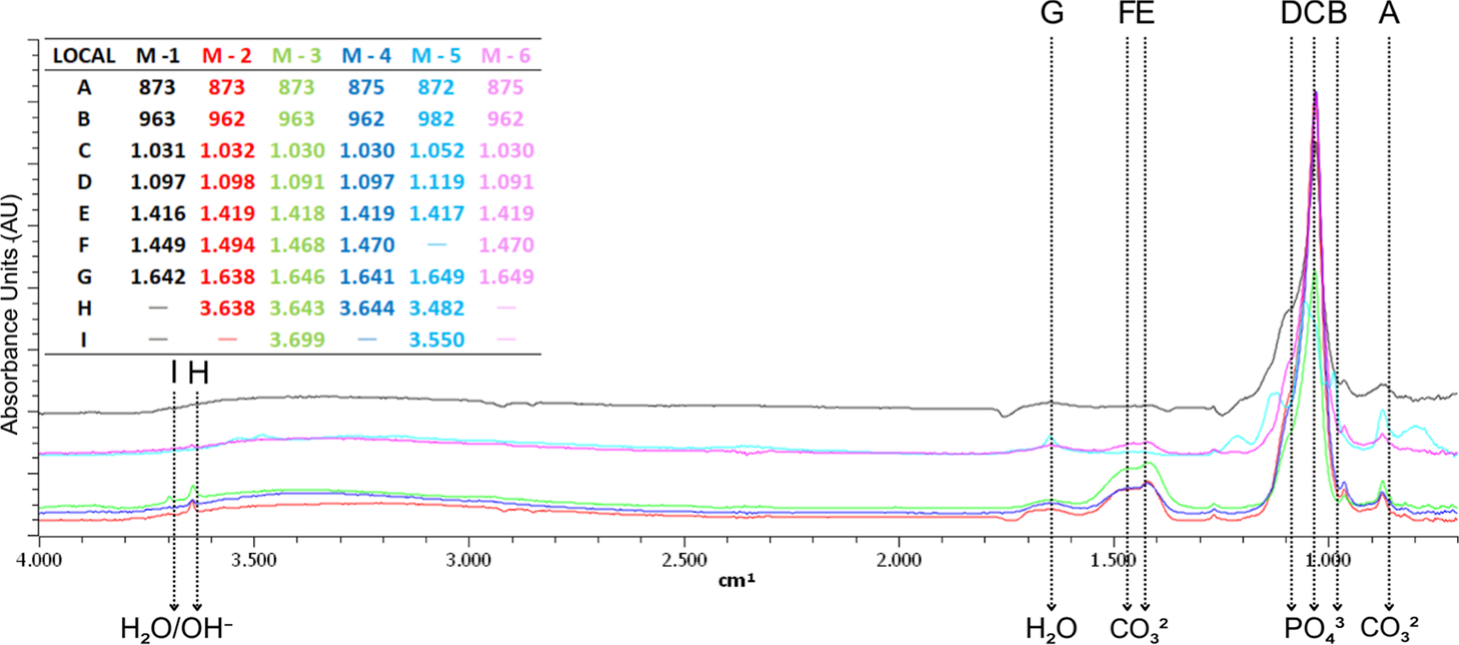
Overlapped FTIR spectra of samples from Methodologies 1–6, with highlighted bands for OH^–^/H_2_O, CO_3_^2–^, and PO_4_^3–^. Peak positions for each methodology are shown in the inset table. Spectral range: 4000–400 cm^–1^; spectral resolution: 4 cm^–1^; number of scans: 32; ATR mode (diamond crystal); measurements performed at room temperature (25 °C).

The same trend is observed between Methodologies 3 and 4 (Figure 5). The heated route (M-3, 90 °C) presents stronger phosphate bands (B–D) and clearer OH^–^ incorporation, reflecting greater long-range order and dehydration of intermediate calcium phosphate phases.^5,21,30^ Methodology 4, performed without heating, exhibits broader phosphate vibrations, reduced intensity, and a barely visible OH^–^ band, consistent with lower crystallinity and greater susceptibility to carbonate substitution.^31^ As a result, M-3 tends to yield a more stable and less soluble HAp, whereas the more hydrated and carbonated M-4 may be suitable for resorbable applications.^34^

The strongest contrast occurs between Methodologies 5 and 6 (Figure 5). Without heating, M-5 presents intense water absorption (∼1649 cm^–1^), irregular carbonate bands, and poorly defined phosphate peaks, indicating the presence of hydrated intermediates such as brushite.^34^ Conversely, Methodology 6 shows sharp phosphate bands, a well-marked ν_3_ shoulder (D), and a defined OH^–^ stretching band, demonstrating higher crystallinity, reduced hydration, and more controlled carbonation.^17,29^

Overall, FTIR analysis provides complementary information to XRD by revealing local chemical features, such as carbonate substitution, hydroxyl deficiency, and structural hydration. While XRD and Rietveld refinement describe long-range crystallographic order and phase composition, FTIR is particularly sensitive to short-range chemical disorder and lattice defects, enabling a more comprehensive interpretation of the effects of thermal treatment on eggshell-derived hydroxyapatite.^10,19,38^

### Scanning Electron Microscopy (SEM)

Figure 6 presents the SEM micrographs for all synthesis methodologies arranged sequentially as follows: Methodology 1 (A–B), Methodology 2 (C–D), Methodology 3 (E–F), Methodology 4 (G–H), Methodology 5 (I–J), and Methodology 6 (K–L). The images illustrate the progressive influence of thermal treatment on agglomeration patterns, porosity, particle coalescence, and nodule development in biogenic hydroxyapatite.

**Figure 6:**
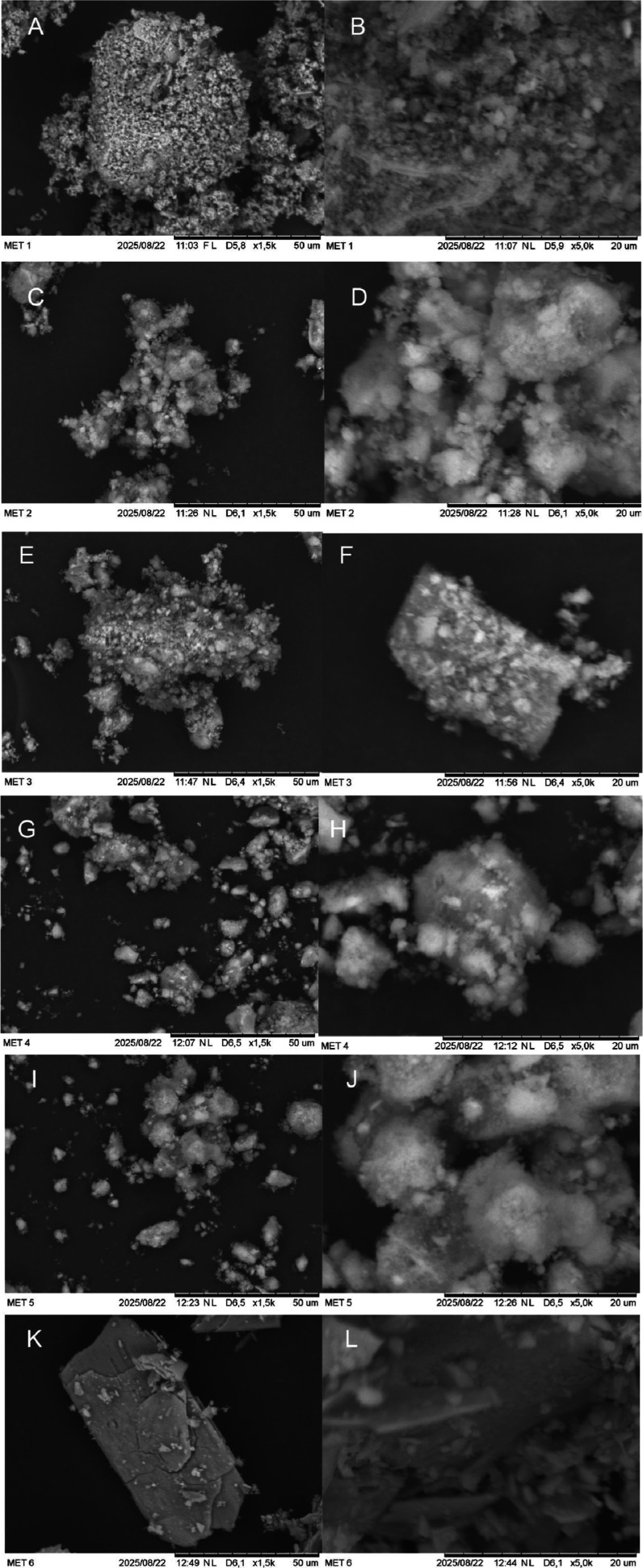
SEM micrographs of HAp synthesized by Methodologies 1–6 (A–L), showing the influence of thermal treatment on agglomeration, porosity, and nodule size. Heated routes produce more compact and crystalline agglomerates, while nonheated routes exhibit fragmented, highly porous structures. Accelerating voltages: 5–15 kV; magnifications: 1500×, and 5000×; secondary electron imaging mode; measurements performed at room temperature (25 °C).

At ∼1500×, Methodology 1 (A) exhibits heterogeneous agglomerates with a cauliflower-like texture and noticeable subpores between fine subunits. This morphology is indicative of limited particle growth, poor packing, and low degrees of necking, consistent with incomplete conversion and low sintering activity. At ∼5000× (B), M-1 displays small and weakly densified nodules (approximately 0.5–1.5 μm), further suggesting restricted crystal growth and a high specific surface area. Such features are frequently reported for cold, low-energy syntheses of calcium phosphates, in which intermediate phases persist and inhibit structural consolidation.^39^

Methodology 2 (C–D) shows clear morphological improvement. At 1500× (C), the agglomerates appear more cohesive, with increased grain-to-grain contact and more extensive necking, reflecting a higher degree of structural reorganization. At 5000× (D), the nodules range from ∼2–4 μm, significantly larger than those of M-1, demonstrating thermally induced coarsening, enhanced crystallinity, and reduced intragranular porosity. This agrees with reports that moderate heating (∼60–90 °C) during wet-chemical precipitation accelerates the transformation of precursor phases into stable HAp and promotes particle coalescence and surface densification.^20^

Methodology 3 (E–F), synthesized at 90 °C, continues this trend. At ∼1500× (E), the agglomerates are compact, with well-defined boundaries and pronounced necking, indicating more complete phase conversion and the onset of superficial sintering processes.^20,39^ At ∼5000× (F), the nodules (∼2–3 μm) are larger and more interconnected, evidencing greater long-range structural order, increased crystallinity, and reduced specific surface area. These features correlate strongly with the improved XRD crystallinity and reduced carbonated intermediates observed for this methodology.

In contrast, Methodology 4 (G–H), performed without heating, produces far less consolidated microstructures. At 1500× (G), the agglomerates are fragmented, with irregular shapes, high macroporosity, and poorly cohesive interfaces—typical of systems with limited nucleation and growth under cold conditions.^5^ At 5000× (H), M-4 displays submicrometric nodules (∼1 μm), weakly interconnected and with rough surfaces, consistent with lower crystallinity, higher solubility, and the persistence of poorly ordered intermediates, as also evidenced by FTIR and XRD.^31^

The most pronounced contrast emerges between Methodologies 5 and 6. The cold route M-5 (I–J) exhibits highly porous agglomerates with coarse, irregular textures and large internal voids. The nodules observed at 5000× (J) remain mostly submicrometric, with diffuse boundaries and significant size dispersion, indicative of amorphous or partially crystalline hydrated phases such as brushite.^34^ This morphology aligns with its incomplete conversion, high water retention, and lower crystalline order.

Methodology 6 (K–L), synthesized under mild heating (70 °C), presents the most advanced microstructural development among all routes. At ∼1500× (K), the agglomerates are more uniform and compact, with well-established necking and reduced porosity. At ∼5000× (L), the nodules reach ∼2–3 μm, are well-defined and tightly interconnected, reflecting superior crystallization, reduced hydration, and enhanced structural stability. These features correlate directly with the XRD results—showing predominant hexagonal HAp—and with the FTIR signature of reduced water content.^20,21,33,39^

In addition to increased agglomerate consolidation, Methodology 6 exhibits plate-like morphological features, indicating anisotropic growth promoted by mild thermal treatment. Such plate-like aggregates are commonly associated with advanced coalescence and surface diffusion during wet-chemical synthesis, reflecting enhanced particle rearrangement rather than simple isotropic aggregation [reference]. The observed structures are typically composed of micrometric subunits (∼2–3 μm), with reduced intergranular porosity compared to nonheated routes. Similar morphologies have been reported for eggshell-derived hydroxyapatite synthesized under controlled thermal conditions and are indicative of improved microstructural organization.^18,35,40^

Overall, SEM analysis reveals a clear structure–processing relationship. Nonheated methodologies (M-1, M-4, and M-5) predominantly exhibit submicrometric agglomerates (<∼1–1.5 μm) with high porosity and weak particle connectivity. In contrast, heated methods (M-2, M-3, and M-6) promote particle coalescence and the formation of more compact agglomerates, with characteristic sizes in the ∼2–4 μm range and reduced pore volume. Among all routes, Methodology 6 shows the highest degree of microstructural development, combining compact agglomerates, plate-like features, and improved homogeneity, consistent with its superior phase composition and crystallinity.^35,40,41^

Despite the systematic comparison presented in this study, certain limitations must be acknowledged. The synthesis routes were evaluated under controlled laboratory conditions by using a fixed Ca/P molar ratio and a single calcium precursor, which may limit direct extrapolation to other biogenic sources or large-scale processes. Furthermore, although several studies report hydroxyapatite formation under low-temperature or nonheated conditions, such routes often require extended aging times, strict pH control, or specific chemical environments to suppress intermediate calcium phosphate phases.^1,2,7^ In the present work, the nonheated methodologies consistently resulted in incomplete conversion, highlighting the sensitivity of chicken eggshell-derived systems to processing conditions.^1,2,7^

Additionally, natural variability in eggshell composition—associated with factors such as animal diet, shell thickness, and mineral heterogeneity—may influence CaO reactivity, hydration behavior, and phase evolution.^1,8^ This intrinsic variability is characteristic of biogenic waste and underscores the importance of process optimization and comparative studies when developing sustainable routes for hydroxyapatite production.

## Conclusion

This study systematically compared six wet-chemical synthesis methodologies for the production of hydroxyapatite from CaO derived from white chicken eggshells. The results demonstrated that nonheated routes led to incomplete formation of the target phase, with the persistence of intermediate calcium phosphate phases and lower crystalline ordering. Among the evaluated methodologies, Methodology 6, conducted at 70 °C, exhibited the best performance, yielding predominantly hydroxyapatite with 88% hexagonal phase and 11% monoclinic phase, nanometric crystallite sizes (≈12.3–33.3 nm), and minimal residual Ca­(OH)_2_ fraction (≈1%). These findings confirm that moderate heating is a decisive parameter for obtaining crystalline and high phase-purity hydroxyapatite from biogenic precursors, without the need for subsequent high-temperature thermal treatments. The proposed route shows potential for biomedical and environmental applications, combining structural performance, reproducibility, and sustainability.
